# Potency of Veterinary Rabies Vaccines Marketed in Sri Lanka

**DOI:** 10.3390/vaccines11050961

**Published:** 2023-05-09

**Authors:** Hasanthi Rathnadiwakara, Mangala Gunatilake, Alexandre Servat, Marine Wasniewski, Jean-Christophe Thibault, Florence Cliquet

**Affiliations:** 1 Department of Physiology, Faculty of Medicine, University of Colombo, Colombo 00800, Sri Lanka; mangalagunatilake@hotmail.com; 2 EU/WOAH/WHO Reference Laboratory for Rabies, OMCL for Rabies Vaccines, 54220 Nancy, France; alexandre.servat@anses.fr (A.S.); marine.wasniewski@anses.fr (M.W.); florence.cliquet@anses.fr (F.C.); 3 Boehringer Ingelheim, 69007 Lyon, France; dr.jcthibault@gmail.com; 4 JC & P Life Science Partner, 04300 Forcalquier, France

**Keywords:** antirabies vaccine, NIH test, rabies, Sri Lanka, potency

## Abstract

Seven brands of veterinary rabies vaccines are commercially available in Sri Lanka, but there is no established procedure to test the potency of the vaccines at the local level, especially prior to their release. The aim of this study was to test the potency of these vaccines using a mouse challenge test in collaboration with the EU/WOAH/WHO Reference Laboratory for Rabies, ANSES-Nancy, France. Based on the European Pharmacopoeia, the inactivated rabies vaccines complied with the mouse potency test if the estimated potency is ≥1.0 IU in the smallest prescribed dose. Among the eight tested vaccines, four single-dose preparations (Rabisin™, Raksharab™, Nobivac™ RL, and Nobivac™ Rabies) were compliant, with potencies of 12 IU/dose, 7.2 IU/dose, 4.4 IU/dose, and 3.4 IU/dose, respectively. Three of the single-dose preparations (Canvac™ R, Defensor™ 3, and Rabies killed vaccine) were not compliant, with potency values <1.0 IU/dose. One multidose preparation (Raksharab™ multidose) had a potency of 1.3 IU/dose, even though the test was not validated. Based on these results, it appears that some rabies vaccine batches that are currently available in the local market do not comply with the mouse potency test. Testing the vaccines’ potency before registration and release to the market appears to be an important step to allow good immunization to animals during pre-exposure vaccination programs.

## 1. Introduction

Rabies is a viral neglected disease that is 100% fatal once a patient becomes ill [1]. Apart from the disease burden and the large number of human deaths reported annually, rabies is responsible for huge economic losses too. This includes dog vaccination costs in excess of USD 130 million and livestock deaths of USD 512 million in rabies-endemic countries [2], such as Algeria, Egypt, and Libya in Africa, Sri Lanka; Pakistan and India in south Asia; Uzbekistan and Georgia in Central Asia; Indonesia, Philippines, and China in southeast and east Asia; and Colombia, Brazil, Bolivia, and Ecuador in South America.

In Asia and Africa, dog bites are the main reason for human deaths following rabies infection [3]. As a rabies-endemic country, Sri Lanka has reported 20–30 human deaths annually due to rabies [4]. Rabies virus has been principally maintained in the dog population (*Canis lupus familiaris*), and the main source of human rabies cases in Sri Lanka has mostly been household pet dogs and stray dogs, the latter being the most involved [5].

The prevention of rabies in animals is fully based on vaccination. The pre-exposure immunization of animal populations, mainly dogs, using effective and safe modern cell culture-derived vaccines, prevents rabies infection and, consequently, transmission to humans [2]. In Western European countries and North America, dogs have been parenterally vaccinated for many years, resulting in the elimination of rabies in dog populations with a concurrent reduction of the burden of human rabies [6]. Following exposure, acute and progressive fatal encephalitis in humans caused by the rabies virus can also be prevented by the timely implementation of postexposure prophylaxis (PEP). This has been demonstrated in many countries and regions in the world [7].

In Sri Lanka, there is evidence of dogs developing rabies despite being previously vaccinated, and rabies cases in humans have been reported after being bitten by vaccinated dogs (personal communication). In other countries, rabies-positive cases in dogs who had been previously vaccinated have been increasingly recognized [3]. This emphasizes the fact that, for the effective control of rabies infection, antirabies vaccines (ARV) should be not only safe but also effective, with the required potency for the successful prevention of virus infection and transmission. There is an upcoming challenge: using ineffective vaccines due to various reasons. The most recent global trend of counterfeit vaccine production is one of those challenges [8]. The increased availability and administration of counterfeit rabies vaccines, especially in PEP, may also result in otherwise preventable rabies deaths in humans [2].

The commonly used test for the potency testing of rabies vaccines is the mouse challenge test, which is based on the National Institute Health (NIH) test, involving double intraperitoneal immunization of mice with different dilutions of the vaccine followed by intracerebral administration of the challenge virus standard (CVS) [9].

In Sri Lanka, as in many countries, human and animal rabies vaccines are currently marketed without any specific registration procedure, considering the results of manufacturers as sufficient for the marketing authorization. A point to consider here is that other than registration of the vaccine brand itself, it is important to control each and every batch that will be marketed in the country because there can be some variations from batch to batch. In this study, the objective was to test the potency (values) of all commercially available veterinary rabies vaccines in Sri Lanka at the time of the study, using the mouse challenge test at the collaborating EU/WOAH/WHO Reference Laboratory for Rabies, ANSES-Nancy, France.

## 2. Materials and Methods

### 2.1. Materials

#### 2.1.1. Test Vaccines

Eight different types of inactivated, adjuvanted rabies vaccines, registered for veterinary use and commercially available in the Sri Lankan market, were tested for their potency using the National Institute of Health (NIH) test. This included six vaccines of single-dose, monovalent or bivalent preparations (Canvac™ R–Dyntec spol. S r.o, Terezin, Czech Republic, Defensor™ 3–Zoetis, Rivonia, Sandton, South Africa, Nobivac™ Rabies and Nobivac™ RL–Intervet International B.V., Boxmeer, Netherlands, Rabies killed vaccine–Komipharm International, Gyeonggi-Do, South Korea, and Rabisin™- Boehringer–Ingelheim (Merial), Germany) and one (Raksharab™- Indian Immunologicals Ltd., Hyderabad, India) with both single-dose and multidose preparations. Seven vaccines were monovalent, and one vaccine was bivalent (rabies and leptospirosis) (Table 1). All these vaccines were directly purchased from their relevant suppliers. Six different brands (Canvac™ R, Defensor™ 3, Nobivac™, Rabies killed vaccine, Rabisin™, and Raksharab™) were purchased from six different suppliers. Nobivac™ Rabies and Nobivac™ RL were purchased from a single supplier. Single-dose Raksharab™ and multidose Raksharab™ were purchased from a single supplier. All eight vaccines were separately registered under the Veterinary Drug Control Authority (VDCA) of Sri Lanka. These vaccines were received in liquid form and stored for 6 months at the Department of Physiology, Faculty of Medicine, University of Colombo, at +4 °C. The storage temperature was regularly monitored, and the cold chain was not broken during this storage period. The vaccines were then shipped to the EU/WOAH/WHO Reference Laboratory for Rabies, ANSES-Nancy, France, for NIH potency testing. Vaccine transportation was also carried out at +4 °C with strict observation. The whole shipping period took three days until arrival. The expiry dates of all vaccines were valid throughout the duration of the study.

#### 2.1.2. Reference Vaccine

The 5th batch of Biological Reference Preparation (BRP No. 5) was obtained from the European Directorate for the Quality of Medicines and Healthcare (EDQM). This preparation, with an assigned titer of 10 International Units (IU) per vial, is a freeze-dried vaccine derived from the Pitman Moore (PM) strain of rabies virus produced in the Nil-2 cell line and inactivated with beta-propiolactone [10]. The BRP No. 5 was stored at −20 °C, and each vial was rehydrated with 1 mL of phosphate-buffered saline (PBS) before use to obtain a solution corresponding to 10 IU/mL.

#### 2.1.3. Animals

Female Swiss/OFI mice (Charles River Laboratory, Les Oncins, France), weighing 13 g–15 g, were used for the analysis. Animals were randomly distributed in groups of 10 mice each (one cage of 10 mice per group) as follows:

1. 4 groups to test the 4 doses of the CVS challenge virus (back titration of the virus).
2. 4 groups to test the 4 doses of the reference vaccine BRP N°5.
3. 4 groups to test the 4 doses of each test vaccine.

#### 2.1.4. Challenge Virus

The CVS-27 used for animal experiments consisted of 20% mouse brain suspension in Dulbecco’s modified Eagle’s medium (DMEM) supplemented with antibiotics and a low percentage (2–5%) of heat-inactivated fetal calf serum. It was stored in 800 µL vials in liquid nitrogen.

### 2.2. Method

#### 2.2.1. NIH Test

The NIH test was performed as described in the European Pharmacopoeia monograph 0451 [11,12]. In brief, four-serial, five-fold dilutions of the vaccine to be examined and the reference vaccine (BRP N°5) were prepared in phosphate-buffered saline (PBS). Each dilution was allocated to a different group of 10 mice, and 0.5 mL of the appropriate dilution was administered via the intraperitoneal route to each mouse on Day 0 (highest dilution administrated first). The initial dilution of the reference vaccine corresponded to 1:1, and the initial dilutions of the test vaccines were 1:4, 1:1, or neat, depending on the potency of the vaccines. Fourteen days after immunization, all mice were anaesthetized (Zoletil^®^, Virbac, France) and challenged with a fixed dose of CVS-27 virus (50 LD50, 0.030 mL) via the intracranial route. The lethal dose 50 (50 LD50) indicated that 0.030 mL of CVS suspension contained the challenge virus that could cause deaths in 50% of the mice when inoculated intracranially. In parallel, 0.030 mL of the challenge suspension and 3-serial, 10-fold dilutions were injected via the intracranial route into each mouse of 4 different groups of 10 unvaccinated mice to perform the back titration of the virus. Animals were monitored daily for the detection of clinical signs of rabies and scored as healthy, ill, or dead. To reduce the duration of animal suffering, humane endpoints were used instead of lethality [11]. Animals were humanely euthanized (by CO_2_ intoxication or cervical dislocation) when typical clinical signs of rabies, corresponding to stage 3 of the disease, were detected.

Any death of an animal occurring during the first four days after the intracerebral inoculation was considered nonspecific and could not be specifically attributed to rabies [9].

#### 2.2.2. Statistical Analysis

The NIH potency test is based on an all-or-none response. Therefore, a parallel-line model with at least 3 points each for the vaccine to be examined and the BRP N°5 was used. Survivorship data at each dilution were submitted to an angular transformation, and linearized dose/response curves of the BRP N°5 and the test vaccines were compared using an in-house database validated against Combistats Software version 4.0, EDQM, Council of Europe, and the potency estimates were determined.

#### 2.2.3. Validation of Potency Tests

In order to make the potency test results valid, the validation criteria needed to be fulfilled [11,13]. The 50% protective dose (PD_50_) should lie between the largest and smallest doses given for both test and reference vaccines. For the challenge virus titration, 0.03 mL of the suspension should contain a minimum of 10 LD_50_. The confidence limits should be between 25% and 400% of the measured potency (*p* = 0.95). Statistical analysis of the CVS titration and the test vaccines should have a significant slope for the dose/response lines. Statistical analysis should show no significant deviations from the linearity of the dose/response lines. There should not be any significant deviation from the parallelism of the dose/response lines of the test and the reference vaccine. Finally, in each group, no more than 2 mice should die within the first 4 days after the challenge.

If all those conditions were fulfilled, the potency of the tested vaccines can be determined, and a vaccine is considered compliant if its estimated potency was equal to or above 1.0 IU/dose based on the European Pharmacopoeia-8.0 [11].

## 3. Results

The results of testing six brands of single-dose preparations and one brand of both single- and multidose preparation are recorded in Table 2. Among the tested vaccines, a total of four single-dose preparations (Rabisin™, Raksharab™, Nobivac™ RL, and Nobivac™ Rabies) were compliant with NIH potency as performed in our conditions. Rabisin™ resulted in the highest potency of 12 IU/dose. Raksharab™ monodose, Nobivac™ RL, and Nobivac™ Rabies had a potency of 7.2 IU/dose, 4.4 IU/dose, and 3.4 IU/dose, respectively. The Raksharab™ multidose preparation had in a potency value of 1.3 IU/dose; however, the test did not meet the validation criteria in which the lower limit was below the threshold of 1.0 IU/mL. Additionally, the estimated potency was just above the threshold (1.3 IU/mL). Two of the single-dose preparations, Defensor™ 3 and Canvac™ R, had potencies just below the threshold. Finally, the Rabies killed vaccine potency was 0.1 IU/dose.

## 4. Discussion

In 2015, the World Organization for Animal Health, the World Organization for Health, and the Food and Agriculture Organization of the United Nations, in partnership with the Global Alliance for Rabies Control, targeted the global goal of zero dog-mediated human rabies deaths by 2030. In their first annual progress report in 2019, they stated that the first objective is to “eliminate rabies by effective use of vaccines, medicines, tools, and technologies” [14]. The strategic plan to eliminate dog-mediated human rabies by 2030 includes the successful control of canine rabies via dog vaccination campaigns [2], as dogs are responsible for the quasi totality of the 60,000 worldwide human deaths each year. To achieve this goal, potent vaccines and medicines are absolutely required. For this purpose, rabies vaccine manufacturers should conduct many in-process quality controls, including identification, purity, sterility, safety, inactivation, and potency [15,16]. The WHO and WOAH recommend that rabies vaccines for human and animal use should be approved by competent authorities and comply with national or international relevant standards [14,16]. The regulatory authorities should also proceed to independent verification of the quality of commercial vaccines in the approval process [14]. This is currently achieved through the use of the NIH test for potency, adopted by the WHO over 50 years ago [17]. This in vivo test was originally developed at the National Institute of Health (Bethesda, MD, USA). It measures the protection conferred by human inactivated rabies vaccines using immunized mice challenged with rabies virus. It is performed by vaccinating mice twice, 7 days apart, followed by an intracranial challenge [16]. The potency test, which was performed for our veterinary rabies vaccines at the EU/WOAH/WHO Reference Laboratory for Rabies, ANSES-Nancy, is the vaccination challenge method on mice described by the European Pharmacopoeia (Ph. Eur) monograph 0451, derived from the NIH test. It was based on single vaccination of mice at Day 0, followed by the intracranial challenge 14 days after the vaccination in groups of ten animals per vaccine dilution in order to meet the first step toward the 3Rs [9,10,15].

There are seven brands of ARV for veterinary use currently marketed in Sri Lanka. In 2016, during the time of planning and obtaining ethics approval for this study, there was no established procedure to test the potency of rabies vaccine batches by the Veterinary Drug Control Authority (VDCA) of Sri Lanka prior to release in the market. Even now, the country does not have established facilities to conduct NIH tests, other serological potency assays, or any quality-control method for veterinary rabies vaccines prior to their use in the fields.

This is the first study evaluating the quality of veterinary vaccines currently in use in Sri Lanka. Among the monovalent vaccines that we tested, Rabisin™, Raksharab™, Nobivac™ RL, and Nobivac™ Rabies were found to be compliant with the NIH potency. The monovalent vaccines Canvac™ R, Defensor™ 3, and Rabies killed vaccine were not compliant with the NIH potency test. These vaccines had potencies below the threshold levels based on the NIH test results. In the test performed with the Rabies killed vaccine, almost all mice died from rabies in every dilution, including the neat dilution. It seems that this vaccine does not confer any protection against rabies infection. The NIH tests of these seven single-dose preparations were validated according to the validation criteria. Even though the Raksharab™ multidose preparation resulted in a potency value of 1.3 IU/dose, the test did not meet the validation criteria because the lower limit of the estimated potency was less than 1 IU/dose, which means we could not make use of the results. We were unfortunately not in a position to repeat the test for this vaccine, as our collaborating laboratory, EU/WOAH/WHO Reference Laboratory for Rabies, ANSES-Nancy, France, had completely discontinued the NIH potency test and replaced it with serological potency assay (SPA), which is a qualitative assay. Even if we repeat the test using SPA, we will only obtain a qualitative result and not an exact level of potency value. Apart from Raksharab™, we also received the multidose preparation of Canvac™ R. The potency test was not undertaken as the batch number was exactly the same as that of the single-dose preparation. This was with the aim of preventing the unnecessary use of animals while respecting the 3Rs principle. In the study performed by Servat et al., 2015 [9], a larger number of different rabies vaccines were tested using the serological potency assay (SPA), by using the modified rapid, fluorescent focus inhibition test (mRFFIT), and the fluorescent antibody virus neutralization test (FAVNt). In the study, among the seven different vaccine groups tested, they had four categories of vaccine potencies: high, medium, and low IUs/dose (close to the threshold of 1 IU/dose) and below the threshold (potency below 1 IU/dose). This indicates that the potencies of the vaccines can vary among each of the vaccine brands.

The variations in antigenic values between vaccines, probably due to the higher or lower antigen concentrations used during in-process production, can be one of the reasons for the differences between the potency values in each of the tested vaccines. The NIH test is a variable method. Sometimes, a 0.3 log variation in the BRP effective dose 50 (ED50) of the day can divide or multiply the potency of the test vaccines by 2. This is because the NIH test is an old method that has not been improved for several decades and therefore suffers from high variability [13]. In addition, this test has some other disadvantages, such as having a high cost, being time-consuming, requiring a large number of animals, and causing significant distress to the animals [13]. EDQM recognized the serological potency assay as an alternative to the mouse challenge, which was adopted through a collaborative study [18]. The SPA is based on the mRFFIT or FAVN test and reduces the number of animals used and the level of pain and distress associated with the procedures in the NIH test, complying with the 3Rs principle [19]. Other techniques, such as ELISA, are currently under development and validation, which would also represent an alternative to the NIH test.

The results of this first study suggest that the assessment of the potencies of ARV appears to be necessary/compulsory before releasing commercial batches to the market, as several brands of vaccines commercialized in Sri Lanka seem to be noncompliant with the mouse potency test. The study will have to be repeated on other batches of the same vaccines to fully validate these first results. Nevertheless, these results are of interest as there is a worldwide concern about the sale of counterfeit or low-quality human vaccines, which leads to the administration of ineffective vaccines. This has made children and adults unknowingly live without protection from this preventable disease [2]. These vaccines can often be sold at more affordable prices than genuine products [20]. This also has been a risk for developing countries where the disease burden is greatest and where vaccines are not readily available and can be prohibitively expensive [2]. It was reported that in 2010, counterfeit human rabies vaccines were administered to more than 1600 people, including children. Studies also reported the risk of counterfeit animal vaccines associated with vaccine storage and vaccine potency. In China, there are records of low-quality rabies vaccines used to vaccinate dogs, which were linked to an increased number of human deaths [3]. Thailand’s Livestock Development Department and Food and Drug Administration reported the discovery of substandard rabies vaccines that had been imported from Spain for animal use in 2016 [2]. All these reported data, together with the results of the present study, confirm the importance of assessing the potency of rabies vaccines before releasing them to the market for vaccinating thousands of dogs.

In Europe, the Official Control Authority Batch Release developed by the European Directorate for the Quality of Medicines and Healthcare (EDQM) is used by several Member States. It consists of the regulatory authorities issuing an official certificate certifying that the batch tested for potency before its marketing complies with the international or national requirements for potency after testing in an independent institute. Such certificates could be recognized by other countries to avoid the use of animals for the test. This batch-control-based system prevents human and veterinary vaccines, which are no longer prequalified, from being sold on the world market. The governments in countries that import human and animal vaccines that are not prequalified should implement a similar system to check vaccine potency on arrival and prior to use. In addition, vaccine manufacturers should be compelled to provide data on the potency values of each exported vaccine batch [2].

Apart from testing the vaccine potency prior to use, there are some other measures that can be adopted by the regulatory authorities to minimize and prevent the use of ineffective and counterfeit animal vaccines. One is the WOAH Rabies Vaccine Bank, which ensures the procurement of high-quality dog vaccines that have been manufactured according to the WOAH international standards [2].

This study reports data on potency tests conducted on specific batches of veterinary rabies vaccines marketed in Sri Lanka, largely used on dogs during rabies vaccination programs. The results demonstrate that three out of seven batches of different vaccines did not fulfill the international requirements for potency. Further testing will be necessary on other batches of these vaccines to confirm the results. The study revealed that some batches of rabies vaccines marketed in the country do not meet the necessary requirements. In addition, we are conducting a study to determine the humoral immunity development conferred by these vaccines on laboratory rabbits and field dogs. Immunization, followed by the measurement of antibody titers, will provide us with data that we can use to re-evaluate and validate the potency test results of the vaccines and allow for a comparison between potency testing and humoral response. As there are no established facilities to conduct NIH tests for veterinary rabies vaccines in Sri Lanka, an alternative could be to evaluate the relative potency of ARVs based on their immunogenicity using a suitable laboratory animal model. However, the WHO recommendation is to evaluate immunogenicity in well-designed field studies [16].

The national authorities of Sri Lanka are currently engaged in the development of a National Strategic Plan for the elimination of dog-mediated human rabies for the years 2022–2026 based on a one-health approach [21]. A major component of this plan will be to increase dog rabies vaccination coverage; hence, this study could be useful for the competent authorities for organizing and implementing mass parenteral vaccination of dogs with potent vaccines. The national government should ensure that the imported vaccines meet the international standard of potency, preventing low-quality vaccines and unregulated products from entering the market. Animal rabies vaccines should be used if they meet the international standards of quality and potency, which will ultimately help to achieve the global vision of zero dog-mediated human rabies deaths by 2030.

## Figures and Tables

**Table 1:** Characteristics of the inactivated rabies vaccines tested in the study.

Vaccines	Manufacturer	Batch Number	Valence	Rabies Virus Strain and (Adjuvants)	Target Species
Canvac R (Single dose 1 mL)	Dyntec spol. S r.o., Czech Republic	010119	Rabies	Inactivated rabies virus strain (Aluminum hydroxide gel)	Dog, cat, fur-bearing animals, cattle, sheep, goat, horse, pig
Defensor 3 (Single dose 1 mL)	Zoetis, South Africa	343374B 364249	Rabies	Strain Pasteur original	Dog, cat, ferret, cattle, sheep
Nobivac Rabies (Single dose 1 mL)	Intervet International B.V., The Netherlands	A508A02	Rabies	Strain Pasteur derivative (Aluminum phosphate)	Dog, cat
Nobivac RL (Single dose 1 mL)	Intervet International B.V., The Netherlands	A138A01	Rabies + Leptospirosis	Strain Pasteur derivative (Aluminum phosphate)	Dog
Rabies killed vaccine (Single dose 1 mL)	Komipharm International, South Korea	58RVKV12	Rabies	Strain Pasteur derivative (Aluminum hydroxide)	Dog, cat, horse, cattle, sheep, goat
Rabisin (Single dose 1 mL)	Boehringer–Ingelheim (Merial), Germany	L465131	Rabies	G52 fixed virus of the Pasteur strain (Aluminum hydroxide)	Dog, cat, horse, sheep, cattle, ferret
Raksharab (Single dose 1 mL)	Indian Immunologicals Ltd., India	01RAB01319	Rabies	CVS * strain (Aluminum hydroxide gel)	Dog, cattle, sheep, camel
Raksharab (Multidose 10 mL)	Indian Immunologicals Ltd., India	03RAB00319	Rabies	CVS strain (Aluminum hydroxide gel)	Dog, cattle, sheep, camel

* Challenge virus standard.

**Table 2:** NIH potency test results of the tested vaccines.

Vaccine	Potency (IU/Dose)
Canvac R	<1
Defensor 3	0.7
Nobivac Rabies	3.4
Nobivac RL	4.4
Rabies killed vaccine	0.1
Rabisin	12
Raksharab	7.2
Raksharab multidose	1.3

## Data Availability

Data is contained within the article.
